# Differential Impact of LPG-and PG-Deficient *Leishmania major* Mutants on the Immune Response of Human Dendritic Cells

**DOI:** 10.1371/journal.pntd.0004238

**Published:** 2015-12-02

**Authors:** Michelle A. Favila, Nicholas S. Geraci, Asha Jayakumar, Suzanne Hickerson, Janet Mostrom, Salvatore J. Turco, Stephen M. Beverley, Mary Ann McDowell

**Affiliations:** 1 Eck Institute for Global Health, Department of Biological Sciences, University of Notre Dame, Notre Dame, Indiana, United States of America; 2 Molecular Microbiology Department, Washington University School of Medicine, St. Louis, Missouri, United States of America; 3 Department of Biochemistry, University of Kentucky College of Medicine, Lexington, Kentucky, United States of America; Queensland Institute of Medical Research, AUSTRALIA

## Abstract

**Background:**

*Leishmania major* infection induces robust interleukin-12 (IL12) production in human dendritic cells (hDC), ultimately resulting in Th1-mediated immunity and clinical resolution. The surface of *Leishmania* parasites is covered in a dense glycocalyx consisting of primarily lipophosphoglycan (LPG) and other phosphoglycan-containing molecules (PGs), making these glycoconjugates the likely pathogen-associated molecular patterns (PAMPS) responsible for IL12 induction.

**Methodology/Principal Findings:**

Here we explored the role of parasite glycoconjugates on the hDC IL12 response by generating *L*. *major* Friedlin V1 mutants defective in LPG alone, (FV1 *lpg1-*), or generally deficient for all PGs, (FV1 *lpg2-*). Infection with metacyclic, infective stage, *L*. *major* or purified LPG induced high levels of *IL12B* subunit gene transcripts in hDCs, which was abrogated with FV1 *lpg1-* infections. In contrast, hDC infections with FV1 *lpg2-* displayed increased *IL12B* expression, suggesting other PG-related/*LPG2* dependent molecules may act to dampen the immune response. Global transcriptional profiling comparing WT, FV1 *lpg1-*, FV1 *lpg2-* infections revealed that FV1 *lpg1-* mutants entered hDCs in a silent fashion as indicated by repression of gene expression. Transcription factor binding site analysis suggests that LPG recognition by hDCs induces IL-12 in a signaling cascade resulting in Nuclear Factor κ B (NFκB) and Interferon Regulatory Factor (IRF) mediated transcription.

**Conclusions/Significance:**

These data suggest that *L*. *major* LPG is a major PAMP recognized by hDC to induce IL12-mediated protective immunity and that there is a complex interplay between PG-baring *Leishmania* surface glycoconjugates that result in modulation of host cellular IL12.

## Author Summary

Leishmaniasis is a group of parasitic diseases caused by intracellular protozoa belonging to the genus *Leishmania*, pathological manifestations ranging from self-healing cutaneous forms to severe visceral infections that result in death. These clinical outcomes are dictated by the *Leishmania* species initiating the infection and are influenced by early responses of host immune cells, which ultimately initiate an IL12 mediated immune response in resolving infections. Like the diseases themselves, the magnitude of IL12 induction in hDCs is *Leishmania*-species and strain specific, where species that elicit visceral disease do not induce IL12, while most cutaneous disease-causing *L*. *major* strains induce robust IL12 responses and confer life-long immunity. The molecular mechanisms that mediate the ability of these innate immune cells to discriminate between pathogens remain elusive and have been primarily investigated in murine model systems. Here we identified *L*. *major* LPG as a major PAMP that induces IL12 in hDCs. Elucidation of this critical component of human immunity to *L*. *major* has ramifications for leishmaniasis vaccine development.

## Introduction

Leishmaniasis constitutes a group of vector-borne parasitic diseases that affects approximately 12 million people worldwide and results in diverse clinical pathologies [1]. The causative intracellular protozoa belonging to the genus *Leishmania*, generally dictate disease outcome in a distinct species-specific manner. Visceral leishmaniasis may result from infection with *Leishmania donovani* parasites that disseminate throughout the body, manifesting into fatal systemic disease if left untreated. In contrast, *Leishmania major*, which is a causative agent for cutaneous leishmaniasis, produces ulcerative lesions localized at the site of sand fly vector inoculation. In the majority of *L*. *major* patients, lesions heal within several months, conferring life-long acquired immunity [2]. Recovery of cutaneous leishmaniasis with a strong immune response can be attributed to early cellular activities that occur following initial entry of the parasites into host cells.

*Leishmania* parasites have evolved mechanisms to survive within host cells and mediate infectivity in sand fly vectors through the interaction of their cellular surface coat molecules. The *Leishmania* surface coat is densely packed with glycosylphosphatidylinositol (GPI)-anchored glycoconjugates, including lipophosphoglycan (LPG), proteophosphoglycans (PPGs), glycosylinositolphospholipids (GIPLs), and glycoprotein 63 (GP63) [3–5]. Together these molecules provide a protective barrier for parasites to persist within the host environment [6]. LPG is one of the most intensely studied *Leishmania* surface molecules, in both the sand fly vector and vertebrate hosts, playing a distinct role in modulating host immune function [7] and even vectorial capacity of various sand fly species [8]. LPG is polymorphic among *Leishmania* species and developmentally regulated [6]. One dominant feature of LPG, the phosphoglycan repeating unit [Gal-Man-P] (PG), contains species-, strain-, and stage-specific modifications usually on the Gal residues [9–13]. The number of PG repeat units almost doubles during metacyclogenesis [14] and LPG is dramatically down regulated in the amastigote stage [15]. Thus, the role of LPG in mammalian infections is limited to the initial period of invasion and establishment of infection by metacyclic promastigotes.

Protective immunity to cutaneous leishmaniasis requires a robust IL12 driven type 1 helper T-cell (Th1) mediated response that produces high levels of interferon-gamma (IFNG), which ultimately promotes anti-microbicidal production of nitric oxide (NO) and reactive oxygen species (ROS) that destroy invading pathogens [16,17]. Dendritic cells (DCs) and macrophages are among the major cell sources of IL12, whose bioactive secretion is dependent upon the covalent linkage between the p40 (IL12B) and p35 (IL12A) subunits [18]. The ability of *Leishmania* to selectively suppress IL12 production, as first established by using murine macrophages [19,20], occurs through the transcriptional inhibition of the *IL12B* promoter [21] and is one immune evasion strategy employed by parasites to establish infection. Phagocytosis of *Leishmania* parasites by murine DCs induces IL12, driving the differentiation of Th1 cells to elicit their effector function [22–27]. The precise role of different DC subsets during murine infection *in vivo* is discordant depending on the *Leishmania* strain utilized, the infection route, and the timing of analysis [28,29]. A role for DCs early in infection has been identified *in vivo*, however, as DCs carrying *Leishmania* antigen produce IL12 within 8 hours following infection [30]. The murine DC IL12 response can be altered depending on the biochemical composition of the parasite surface, as evidenced by a study demonstrating that infection with *L*. *major* LV39c5 *lpg2* ^−^, a mutant that lacks phosphoglycan (PG)-containing molecules and other LPG2-dependent metabolites [31], induced IL12B in bone marrow derived mouse DCs (BMDCs) co-stimulated with anti-CD40 and IFNG [32]. This effect along with the long-term persistence of these parasites likely account for why vaccination with these LV39c5 *lpg2* ^−^ parasites protects mice against *L*. *major* wild type (WT) challenge [33].

Remarkably, hDCs exhibit a dynamic range in IL12 production in response to *Leishmania* infection that is largely dependent upon the nature of the infecting species or strain. *L*. *donovani* fails to elicit IL12, whereas a general induction of IL12 is observed during *L*. *major* infections [34]. However, IL12 production also varies across *L*. *major* strains. Strains LV39 and SD do not induce IL12, whereas Friedlin V1 (FV1), IR173, IR176, and CC-1 strains elicit high levels of IL12 [34,35]. These differences are not well-correlated with LPG structural polymorphisms, as *L*. *major* LV39cl5 bears a highly poly-galactosylated LPG [36], while *L*.*major* SD synthesizes an unsubstituted LPG similar to that of *L*. *donovani* [37]. Several groups have reported differences in lesion pathology following *in vivo* infection with these same *L*. *major* strains. For example, *L*. *major* FV1 infected C57BL/6 mice develop lesions that eventually heal over time, whereas mice infected with *L*. *major* SD produce non-healing lesions [38]. BALB/C IL4RA knockout mice are resistant to *L*. *major* IR173 strain but susceptible to *L*. *major* LV39 strain [39]. Moreover, while *L*. *major* FV1 strain infected BALB/C mice quickly develop lesions, *L*. *major* LV39c5, a clonal derivative of the LV39 strain, elicits slower lesion development. Hybrid crosses of *L*. *major* FV1 x LV39c5 segregate at a 1:1 ratio into “fast” or “slow” virulence progeny [40]. These differential host responses to variant intra-species strains of *L*. *major* have important implications for the parasite strain-specific factors that could dictate disease persistence versus healing and induction of immunity.

In this study, we focus on elucidating whether parasite surface molecules are associated with the robust cytokine response observed in hDCs using the ‘high-IL12 inducing’ *L*. *major* FV1 strain. We generated parasite mutants lacking LPG alone, as done previously with the ‘low-IL12 inducing’ *L*. *major* LV39c5, through inactivation of the LPG1 galactofuranosyl transferases required for LPG core synthesis. Mutants generally lacking in all PG-containing structures were generated through inactivation of the Golgi GDP-mannose nucleotide sugar transporter gene, *LPG2* [31]. This approach is powerful for probing the role of LPG as it allowed us to assess the impact of LPG deficiency in the context of the parasite, rather than through exogenous and relatively artificial routes. A second advantage is that multiple mutants provided a means to discriminate between LPG effects and those of molecules that bear structures related to or shared with those found in LPG. Notably, the PG repeating units present on LPG also are abundant on secreted molecules, such as acid phosphatases and other PPGs, which can be anchored to the parasite surface through glycosylphosphatidylinositol (GPI). Inactivation of *LPG1* results in a parasite lacking LPG alone but otherwise normal in GIPL and PPGs levels [41].

Our results demonstrate that hDC infection with the LPG-null *L*. *major* FV1 *lpg1* ^−^ mutant resulted in significantly diminished *IL12B* mRNA, relative to FV1 WT parasites, indicating that LPG is essential for stimulating host IL12 production. However, the PG-null *L*. *major* FV1 *lpg2* ^−^ mutant infected DCs exhibited an increase in *IL12B* expression, suggesting that PGs and/or other LPG2-dependent metabolites may suppress IL12 induction. These results suggest that *L*. *major* parasites balance stimulatory and inhibitory effects on the host cells to establish infection.

## Materials and Methods

### Ethics statement

The study protocol was approved by the University of Notre Dame Institutional Review Board in compliance with all applicable Federal regulations governing the protection of human subjects (Human Subjects Assurance #M1262). The research was deemed exempt under exemption #4. The samples were purchased from Central Indiana Regional Blood Center, Indianapolis, IN and no identifying information was provided.

### Dendritic cell generation and infection

Monocytes were isolated from healthy human donor buffy coats (Central Indiana Regional Blood Center, Indianapolis, IN) by enriching for CD14^+^ cells using a magnetic bead separator (AutoMACs, Miltenyi Biotech systems, Germany). Monocytes from each donor were cultured in 6-well plates at a concentration of 10^6^ cells/2ml of RPMI-complete media (10% heat-inactivated FBS, 2mM l-glutamine 100U/ml, 1% penicillin/streptomycin) and supplemented with recombinant human IL4 (40U/ml, Peprotech, NJ) and granulocyte-macrophage colony-stimulating factor, GMSCF (1000U/ml, Peprotech, NJ) on days 0, 3, and 6 to allow differentiation into immature DCs. Cells were harvested, washed one day before infection to remove any residual cytokines, and assessed for DC marker CD1A to confirm a homogenous population of immature DCs. All parasite strains were cultured at 26°C without CO_2_ in M199 medium containing 10% heat-inactivated FBS [42]. Metacyclic promastigotes were isolated according to previously described methods [43] and opsonized by treatment with 5% human serum for 30 min at 37°C. DCs were then infected at a concentration of 10 parasites per 1 DC in RPMI-complete media. As we previously demonstrated that the peak of *IL12B* expression occurs at 8 hours post *L*. *major* infection [44] and to avoid the complication that mutant parasites might be degraded at later time points as previously observed [31,41], samples were typically harvested at 8 hours post-infection. For kinetic analyses we focused on the early time points following infection (2, 4, 8, or 24 hours). Cytospins were prepared at the conclusion of each experiment and Diff-quick stained (Fischer Scientific, Pittsburgh, PA) for visual analysis by light microscopy. Uninfected and infected DCs (100 total) were counted to calculate the infection rate (% infected DCs) and the parasite indices (# parasites per 100 cells) for each infection sample. All parasite and human cell cultures tested negative for mycoplasma (PCR detection, Takara) and tested below the limits of detection for endotoxin (<0.25U/ml) (Limulus Amoeboctye Assay, Endosafe, Charleston, NC).

### Generation of *L*. *major* FV1 *lpg1* ^−^ and *L*. *major* FV1 *lpg2* ^−^ mutants and complemented lines

*Leishmania major* strain Friedlin clone V1 (MHOM/IL/81/Friedlin) and *L*. *donovani* strain 1S (MHOM/SD/62/1S) were grown in M199 medium containing 10% heat-inactivated FBS [45]. Methods for electroporation of logarithmic phase promastigotes and plating on semisolid media to obtain clonal lines were as described previously [46].

*L*. *major* FV1 *lpg1* ^−^ mutants were obtained by a gene disruption strategy, in which autonomous drug resistance cassettes were inserted within the *LPG1* coding region [41]. The methods and constructs used were the same as in the prior study generating the *L*. *major* LV39c5 *lpg1* ^−^ mutants [41]. In the first round, plasmid B2947 DNA was digested with restriction enzymes XhoI and HindIII to yield the *LPG1*::*HYG* targeting construct, conferring selective resistance gene to hygromycin B (hygromycin phosphotransferase). 10μg of DNA was used for electroporation and parasites were plated on semisolid medium containing 50μg/ml of hygromycin B. Clonal parasite lines were obtained at typical frequencies and screened for the presence of the expected heterozygous *LPG1* and *LPG1*::*HYG* insertion by PCR (S1 Fig, S1 Table). Several clones were inoculated into susceptible BALB/C mice (10^7^ stationary phase, footpad) and recovered after 1 month; such mouse passaged lines are designated as ‘M1’. These heterozygotes underwent a second round of transfection; electroporating 10μg of *LPG1*::*PAC*, conferring a selective resistance gene to puromycin (puromycin acetyltransferase), derived from BamHI digestion of plasmid B2949, and followed by plating parasites on semisolid media containing 50μg/ml hygromycin B and 30μg/ml puromycin. Clonal lines bearing disruptions in both *LPG1* alleles, and lacking unmodified *LPG1* (^*LPG1*::*HYG/^LPG1*::*PAC*), were identified by PCR analysis and confirmed by Western blot analysis and agglutination tests. Several clones were inoculated into susceptible BALB/C mice (10^7^ stationary phase, footpad) and recovered after 1 month (M1). For simplicity, these lines are referred to as FV1 *lpg1* ^−^. To generate complemented ‘add back’ lines, several FV1 *lpg1* ^−^ clonal lines were electroporated with the *LPG1* expression plasmid pSNBR-*LPG1*::*NEO* (B3340), conferring an episomal selective resistance gene to the aminoglycoside antibiotic G418 via expression of the neomycin phosphotransferase gene *NEO*, and clonal lines were recovered by plating on semisolid media containing 50μg/ml HYG, 30μg puromycin, and 12μg/ml of G418. Successful transfection was established by PCR tests and restoration of LPG expression by western blot, and agglutination tests. Formally, the genotype of such lines is (^*LPG1*::*HYG/^LPG1*::*PAC/+pSNBR-LPG1*), which for simplicity is referred to as FV1 *lpg1* ^−^ */+LPG1*. Sibling clonal lines displayed similar phenotypes and one representative FV1 *lpg1* ^−^ line (cl2.10, M1), and its complemented offspring (cl2.10 AB3, M1), designated FV1 *lpg1* ^−^ */+LPG1* were used in the experiments.

*L*. *major* FV1 *lpg2* ^−^ mutants were obtained by a gene replacement strategy; where the drug resistance gene ORFS replaced the *LPG2* coding region. In the first round, plasmid B3950 was digested with XhoI I, yielding the *LPG2*::*HYG* targeting construct; 10μg was used for electroporation and cells were plated on semisolid medium containing 50μg/ml of hygromycin B. Clonal lines were obtained at typical frequencies and screened for the presence of the expected heterozygous *LPG2* and *LPG2*::*HYG* insertion by PCR (S2 Fig, S1 Table). Several clones were inoculated into susceptible BALB/C mice (10^7^ stationary phase, footpad, M1). These heterozygotes underwent a second round of transfection, electroporating 10μg of *LPG2*::*SAT*, conferring a selective resistance gene to nourseothricin (streptothricin acetyltransferase), derived from XhoI, HindIII digestion of plasmid B6598, followed by plating on semisolid media containing 50μg/ml hygromycin B and 100μg/ml nourseothricin. Clonal lines bearing disruptions in both *LPG2* alleles and lacking unmodified *LPG2* (Δ*LPG2*::*HYG/* Δ*LPG LPG2*::*SAT*) were identified by PCR analysis, and confirmed by Western blot analysis and agglutination tests. For simplicity, these lines will be referred to as FV1 *lpg2* ^−^. To generate complemented ‘add back’ lines, several FV1 *lpg2* ^−^ clonal lines were electroporated with the *LPG2* expression plasmid pXG-*LPG2*::*NEO* (B4296) and clonal lines recovered by plating on semisolid media containing 50μg/ml HYG, 100μg/ml SAT, and 15μg/ml of G418. Successful transfection was established by PCR tests and restoration of LPG and the PPGs region expression by western blot, and agglutination tests. Formally, the genotype of such lines is (Δ*LPG2*::*HYG/* Δ*LPG LPG2*::*SAT/+pXG-LPG2*), which for simplicity is referred to as FV1 *lpg2* ^−^ */+LPG2*. Sibling clonal lines displayed similar phenotypes and one representative FV1 *lpg2* ^−^ line (cl6.1A, M1), and its complemented offspring (cl6.1A AB15, M1), designated FV1 *lpg2* ^−^/+LPG2 were used in the experiments.

### Gene replacement plasmid generation

Plasmid B6598 was generated by a fusion PCR strategy. Briefly, the 5’*LPG2* flanking sequence, 3’*LPG2* flanking sequence, *LPG2* ORF, and selected drug marker, *SAT* ORF were amplified by PCR and inserted into the pGEM-T-Easy vector by TA cloning according to manufacturer’s instruction (Promega, Madison, WI) and transformed into *E*. *coli*. Its structure was confirmed by DNA sequencing. The primers used for constructing B6598 are provided in S1 Table.

### Western blot

For Western blot analysis of PG-containing molecules, parasites were grown to logarithmic phase and harvested for cell lysate preparation in 4X Lamelli buffer (50 mM Tris-HCl pH 6.8, 2% SDS, 10% Glycerol, 1% 2-mercaptoethanol, 12.5 mM EDTA, and 0.02% Bromophenol Blue). Samples were separated on 10–12% SDS-PAGE gels at a concentration of (3.5x10^6^ cells/well) and transferred onto methanol activated nitrocellulose membrane for 3 hrs at 60V, 4°C. Ponceau staining was performed to assure macromolecule transfer prior to blocking in 5% milk overnight. Membranes were stained with primary mouse monoclonal anti-sera WIC79.3 antibody (1:1000), recognizing galactosylated Gal-Man-P repeats on LPG, and detected using a goat anti-mouse HRP conjugated secondary antibody (1:5000) (Invitrogen, Carlsbad, CA). Membranes were developed using West-Pico detection solution assay (Thermo Scientific, Rockford, IL) and an X-ray film developer.

### LPG purification

LPG was isolated from 10^9^ *L*. *major* FV1 metacyclic promastigotes as previously described, with minor modification [47,48]. Cellular membranes were disrupted by sonicating pelleted cells suspended in a cold chlorform:methanol:water (1:2:0.8) solution, centrifuged (5000rpm, 10min, 4°C), and the top de-lipidated layer containing the majority of GIPLs and phospholipids was removed. The remaining insoluble material was quick-dried under stream of N_2_ and further extracted with two rounds of 9% 1-butanol extraction to release LPG molecules into the top aqueous layer. Hydrophobic interaction chromatography was performed to purify LPG molecules from the *Leishmania* surface coat. Briefly, LPG-containing butanol extracts were pooled and added to a 20% Octyl-Sepharose column that was pre-equilibrated with (5% proponal, 1M ammonium acetate). A desalting gradient (5%-60%) was applied to the column to elute LPG fractions utilizing the fraction collector, (BioRad Fraction Collector, Model 2128). LPG was detected by thin layer chromatography (TLC) and quantified by phenol sulfuric assay. Sample fractions were spotted on silica containing TLC plate. Glycan determinants were visualized by spraying the plate with orcinol (0.5mg/ml in 95% ethanol), dried, and sprayed with 75% sulfuric acid. All LPG containing fractions were pooled and dried in speed-vacuum at room temperature. Lyophilized LPG was resuspended in water and quantified by a colorimetric phenol-sulfuric assay [49]. Purification of LPG molecules was confirmed by a standard Stains-All protocol. Briefly, 5–10μg of LPG was boiled in 2X Loading Dye and loaded onto 10% SDS PAGE gel, running at 140V (room temperature). Gels were fixed in 25% 2-propanol and stained with stains-all solution (Fluka Analytical, Switzerland) containing 10% formamide, followed by destaining with 40% ethanol. Bands were visualized under white light, based on the observation that LPG molecules give rise to a blue colored complex (wavelength– 649nm) [50]. WIC79.3 western blot analysis was utilized to confirm LPG purification. Lyophilized purified LPG was resuspended in serum free RPMI and a titration of LPG (0.5μg, 1μg, and 10μg) was used for the hDC infection assay.

### Quantitative real-time polymerase chain reactions

Relative levels of human gene transcripts were determined by qRT-PCR. Total RNA from uninfected or *Leishmania*-infected DCs was isolated using an RNeasy kit (Qiagen, Valencia, CA) and 1μg of RNA per infection sample was used to generate cDNA using SuperScript III Synthesis (Invitrogen, Carlsbad, CA) according to manufacturer’s instructions. For analysis of *IL12B*, *IL12A*, *IRF1*, *IRF8*, *TNF*, *IL10*, *IL1B*, *SOCS3*, *TNFAIP3*, and *HPRT* (hypoxanthine-guanine phosphoribosyltransferase) mRNA expressions, qRT-PCRs were conducted utilizing SYBR Green PCR Master Mix (Applied Biosystems by Life Technologies, Carlsbad, CA) according to manufacturer’s protocol and detected with an ABI 7900HT Fast Real-Time PCR System (Applied Biosystems by Life Technologies, Carlsbad, CA). All human primer sequences were designed by Integrated Design Tools (IDT) and used at a concentration of 5μM per reaction (S2 Table). For select analysis of *IL12B*, *IL12A*, and *GAPDH* (glyceraldehydes 3-phosphoate dehydrogenase) mRNA expressions, PCR reactions were setup employing TaqMan pre-developed assay kits (Life Technologies, Foster City, CA) and determined using an ABI 7500 Real Time PCR System (Applied Biosystems, Foster City, CA). For each gene, relative numbers of mRNA copies were determined by the ΔΔC_T_ method [42].

### Microarray expression profiling

Total RNA was isolated 8 hours post-infection from four additional donors’ uninfected monocyte-derived DCs and DCs infected with *L*. *major* FV1 WT, FV1 *lpg1* ^−^, FV1 *lpg1* ^−^/+*LPG1*, FV1 *lpg2* ^−^, and FV1 *lpg2* ^−^/+*LPG2* using RNeasy kits (Qiagen, Valencia, CA). RNA 6000 Nano kits (Agilent Technologies, Santa Clara, CA) were used to determine total RNA integrity on a Bioanalyzer 2100 instrument (Agilent Technologies, Santa Clara, CA). 25ng of high quality RNA was converted to double stranded cDNA using a TransPlex Complete Whole Transcriptome Amplification kit (Sigma-Aldrich, Saint Louis, MO). RNA degradation, double stranded cDNA purification, and cDNA precipitation was conducted following NimbleGen Gene Expression Array user’s guide protocols (Roche-NimbleGen, Madison, WI). A Nanodrop ND-2000 (Thermo Fisher Scientific, Waltham, MA) was used to determine total RNA and double stranded cDNA concentrations. Sample cDNAs were Cy3-labeled using NimbleGen Single Color Labeling Kit (Roche-NimbleGen, Madison, WI) per manufacturer's recommendations. Labeled cDNAs were hybridized to 12-plex NimbleGen *Homo sapiens* Expression Arrays (platform GPL16025), featuring 140,096 probes, representing 21,269 genes and transcripts, using Hybridization LS and Wash Buffer Kits (Roche-NimbleGen, Madison, WI) per manufacturer's recommendations. Image acquisition of arrays was performed using a NimbleGen MS 200 Microarray Scanner (Roche-NimbleGen, Madison, WI), at a 2 micron resolution. NimbleGen array image data were processed using NimbleScan version 2.5 (Roche-NimbleGen, Madison, WI) to extract intensity values for each gene. NimbleScan software automates the pre-processing of NimbleGen microarray image data, including identifying the location of each probe, extraction of intensity data from the image, background correction, and obtaining expression summary values for each gene using a probe-level summarization robust multi-array average method (RMA). Probes with intensity values greater than twice that of background were retained for downstream analysis. Log_2_ normalized expression ratios for each gene were calculated between infected samples and paired uninfected samples. Z-scores were calculated between infected and uninfected samples as previously described [51]. Briefly, Z-score = (log_2_(infected intensity value/inter-quartile mean of uninfected intensity values)_Gi_−average(log_2_(infected intensity value/ inter-quartile mean of uninfected intensity values)_Gi…Gn_) / standard deviation(log_2_(infected intensity value/ inter-quartile mean of uninfected intensity values)_Gi…Gn_). An absolute Z-score value of 1.96 may be inferred as significant (p<0.05) [51]. Complete array data generated in this study are accessible at the NCBI Gene Expression Omnibus database (accession GSE59766). Gene expression data of RMA normalized raw microarray probe hybridization fluorescence values, where at least one sample value was twice that of background resulted in 12,911 genes.

### Functional enrichment analyses

Genes that displayed significant differential expression from FV1 WT, FV1 *lpg1* ^−^, or FV1 *lpg2* ^−^ samples compared to uninfected samples on NimbleGen microarrays were fed into the Short Time-series Expression Miner (STEM) program [52,53]. Briefly, log_2_ ratio values for each of four donors were loaded into the program as repeated data, where FV1 WT data represented a “time point 1”, FV1 *lpg1* ^−^ data represented “time point 2”, and FV1 *lpg2* ^−^ data represented “time point 3”. The datasets were clustered using the STEM clustering method with minimum correlation values of 0.6. The genes from the resultant model expression profile containing *IL12B* were used for downstream enrichment analysis in the Web-based Gene Set Analysis Toolkit (WEBGESTALT) [54,55] with a simple list of 233 official gene symbols as input. KEGG Pathway enrichment was conducted on that list of genes with similar expression profiles to that of *IL12B* using the following parameters: protein-coding EntrezGene database as a reference set and a hypergeometric test with Benjamini and Hochberg multiple test adjustments. Pathways with an adjusted p-value < 0.01 and a minimum of three genes found were considered significant. The same list of gene symbols was input to The Database for Annotation, Visualization and Integrated Discovery (DAVID) v6.7 Functional Annotation Tool [56] and transcription factor binding sites for each gene were identified using protein interaction enrichment. The annotations were cross-referenced to report the most common transcription factor binding sites found in the *IL12B* gene and genes with *IL12B*-like expression between DC samples infected with *L*. *major* FV1 LPG mutants.

### Statistical analysis

All statistical tests were performed using Graph Pad Prism version 5.0 (Graph Pad Software, San Diego, CA). Statistical analysis was performed using Log2 transformed ΔΔC_T_ values using a paired Student’s T-test. Differences were considered significant at p<0.05.

## Results

### Differential IL12 response in hDCs is *L*. *major* strain and developmental stage specific

**Fig 1:**
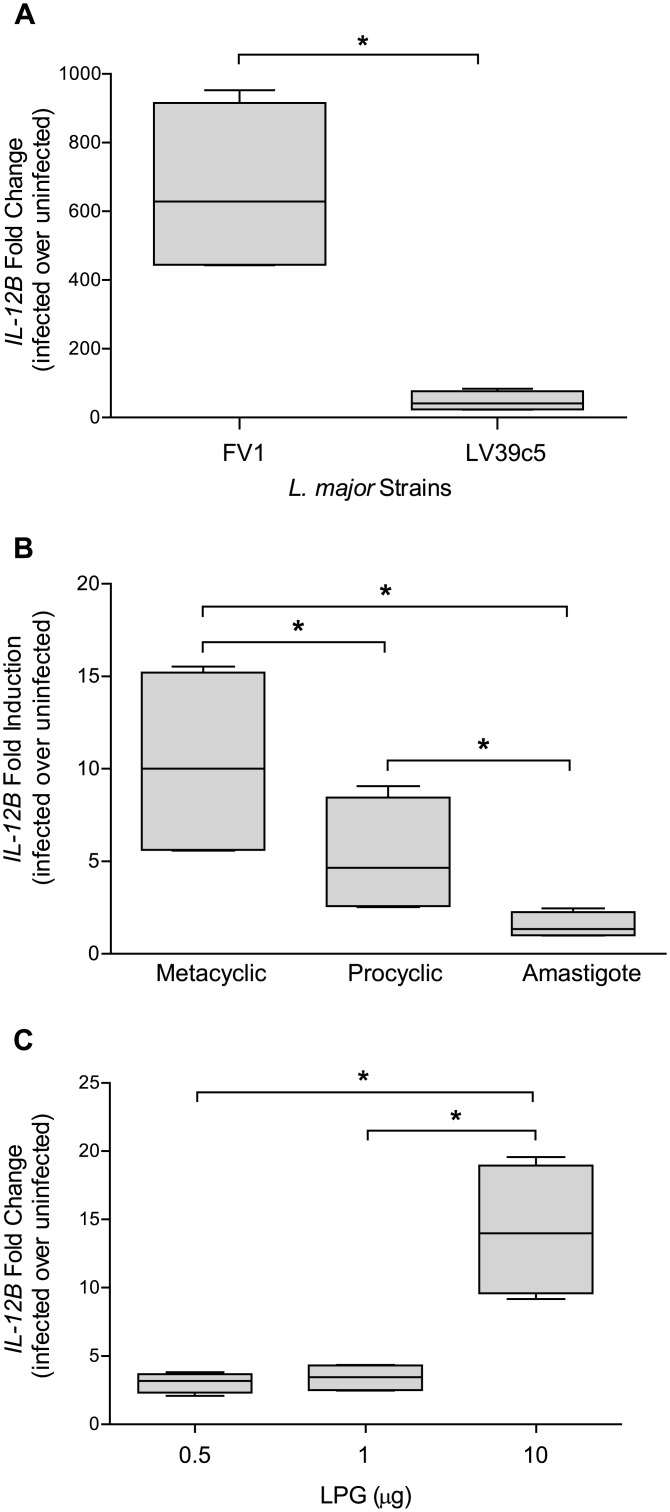
*L*. *major* strain FV1 metacyclic promastigotes and LPG stimulate *IL12B* expression. (**A**) Human DCs were infected with *L*. *major* FV1 or *L*. *major* LV39c5 parasite strains (n = 4 donors). After 8 hours, RNA was extracted from infected hDCs for cDNA generation and analyzed for *IL12B* expression by qRT-PCR. All values were significantly greater than uninfected. (**B**) Human DCs were infected with *L*. *major* FV1 metacyclic promastigotes (metacyclic), procyclic promastigotes (procyclic) or amastigotes (n = 3 donors). After 8 hours, RNA was extracted from infected hDCs for cDNA generation and analyzed for *IL12B* expression by qRT-PCR. All values were significantly greater than uninfected, except for amastigote infections. (**C**) Human DCs were exposed to different concentrations of LPG (0.5, 1, and 10 μg), derived from *L*. *major* FV1 metacyclic promastigotes (n = 4 donors). After 8 hours, RNA was extracted from infected hDCs for cDNA generation and analyzed for *IL12B* expression by qRT-PCR. Fold change was calculated utilizing the ΔΔC_T_ method and depicted as fold change over uninfected samples. Box plots display the median value (line), the interquartile range (box), and Tukey whiskers encompassing data within 1.5 fold of the interquartile range. *Statistical significance as compared to uninfected control, (p<0.05). All values were significantly greater than uninfected.

First, we confirmed that the *IL12B* mRNA expression in hDCs infected with *L*. *major* strains FV1 WT was greater than DCs infected with LV39c5 WT. We demonstrated that *L*. *major* FV1 induced approximately 15-fold greater amounts of *IL12B* than *L*. *major* LV39c5 (Fig 1A) at 8 hours post infection, the optimal time for peak *IL12B* mRNA expression following *L*. *major* infection [44]. These data support previous work that illustrated that the hDC IL12 response is strain-specific, and also that infection with *L*. *major* FV1 promotes a high induction of IL12 and that LV39c5 is similar to the LV39 strain tested previously [34]. We also confirmed that the increased *IL12B* expression observed during *L*. *major* FV1 WT infections were significantly associated with the infective metacyclic promastigote stage, whereas smaller effect was observed with the non-infective procyclic promastigote stage, and no response was elicited by amastigotes (Fig 1B). These data are consistent with prior studies indicating that IL12 induction depends on the life cycle stage of *Leishmania* parasites [57,58].

### Purified LPG induces *IL12B* expression

To determine whether the enhanced *IL12B* production observed following infection with *L*. *major* FV1 metacyclic promastigotes was an LPG-dependent response, we first assessed the role of purified LPG on the hDC *IL12B* response. Human DCs were cultured in the presence of varying amounts of purified metacyclic *L*. *major* FV1 LPG for 8 hour and then assessed for *IL12B* expression. At lower concentrations (0.5, 1 μg), LPG induced a slight increase over uninfected samples, while at a higher concentration (10 μg) a significant 15-fold induction of *IL12B* mRNA was observed (Fig 1C), indicating that LPG alone is capable of stimulating IL12B production. Albeit to a lower level than what is observed with *L*. *major* LPG, purified *L*. *donovani* LPG induced a significant increase in *IL-12B* expression in 2 out of 3 donors (S5 Fig). Due to variation amongst the human donors, however, this difference was not statistically significant.

### Generation of *L*. *major* FV1 LPG- and PG-null mutants and complemented lines

To probe the role of LPG and related PGs in host cell IL12 responses in the context of a *Leishmania* infection, we generated parasites lacking LPG alone (FV1 *lpg1* ^−^) or all PGs (FV1 *lpg2* ^−^) (Table 1). As *L*. *major* strain FV1 is disomic for chromosomes 25 and 34 bearing *LPG1* and *LPG2* respectively, two rounds of gene targeting were required to generate null mutants (S3A and S3B Fig). PCR tests confirmed the loss of *LPG1* (S1B Fig) and *LPG2* (S2B Fig) ORFs in the FV1 *lpg1* ^−^ and FV1 *lpg2* ^−^ mutants, respectively. Similarly, PCR tests confirmed the generation of the planned genetic alterations for the *LPG1* disruption (FV1 *lpg1* ^−^) (S1C Fig) and the *LPG2* replacement (FV1 *lpg2* ^−^) (S2C Fig). Complemented ‘add back’ lines were generated by introducing episomal constructs expressing the *LPG1* or *LPG2* genes into their respective null mutants (S3A and S3B Fig, bottom), which were confirmed by PCR and drug sensitivity tests. Western blot analysis with an anti-PG anti-sera (WIC79.3) showed that LPG expression alone was lost in the FV1 *lpg1* ^−^ mutant (S3C Fig, lane 6) and restored in the complemented FV1 *lpg1* ^−^ */+LPG1* line (S3C Fig, lanes 4 and 5). Similarly, Western blot analysis with WIC79.3 verified the absence of both PPGs and LPG in the FV1 *lpg2* ^−^ mutant (S3C Fig, lane 2), and their restoration in the complemented FV1 *lpg2* ^−^ */+LPG2* line (S3C Fig, lane 3).

**Table 1:** Formal names for *L*. *major* FV1 LPG and PG null mutants and add back lines.

*L*. *major* strain	^*a*^Alleles	Loss of function
FV1 WT	*LPG1/LPG1; LPG2/LPG2*	
FV1 *lpg1* ^−^	*^LPG1*::*HYG/^LPG1*::*PAC*	LPG biosynthesis
FV1 *lpg1* ^−^ */+LPG1*	*^LPG1*::*HYG/^LPG1*::*PAC + LPG1*::*NEO*	
FV1 *lpg2* ^−^	*ΔLPG2*::*HYG/ΔLPG2*::*SAT*	PG biosynthesis
FV1 *lpg2* ^−^ */+LPG2*	*ΔLPG2*::*HYG/ΔLPG2*::*SAT+LPG2*::*NEO*	

^a^A ^ denotes gene disruption and a ^Δ^ denotes gene replacement

### *L*. *major* FV1 LPG required for robust IL12 responses in hDCs

**Fig 2:**
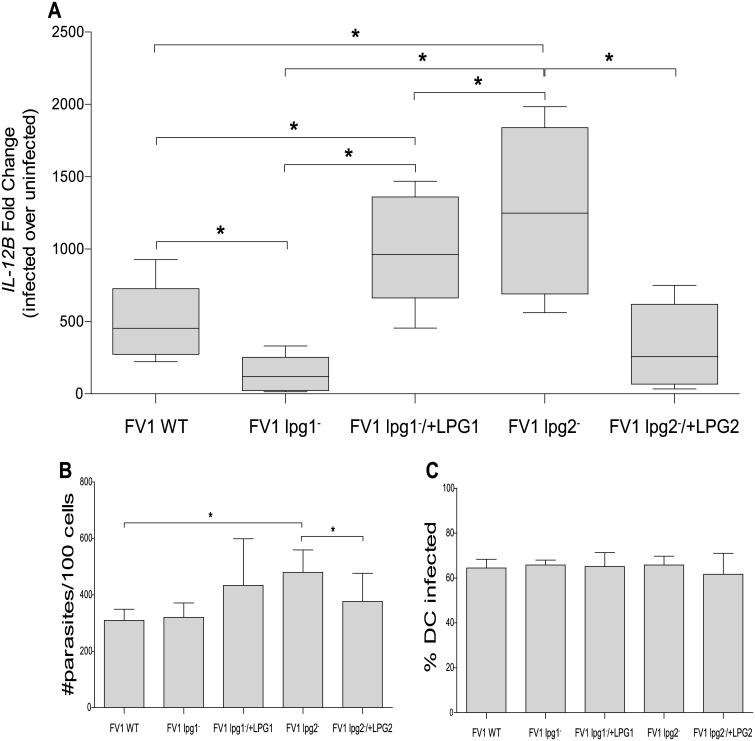
*L*. *major* FV1 *lpg1* ^−^ and FV1 *lpg2* ^−^ modulate the *IL12B* response in hDCs. Human DCs (n = 9 donors) were infected with *L*. *major* FV1 parasites: *L*. *major* FV1 (WT), LPG null mutant (FV1 *lpg1* ^−^), LPG add back (FV1 *lpg1* ^−^/+*LPG1*), PG null mutant (FV1 *lpg2* ^−^), or PG add back (FV1 *lpg2* ^−^/+*LPG2*). (**A**) At 8 hours post infection, *IL12B* expression was measured by qRT-PCR. Fold changes were calculated using the ΔΔC_T_ method and are represented as fold change over uninfected samples. Box plots display the median value (line), the interquartile range (box), and Tukey whiskers encompassing data within 1.5 fold of the interquartile range. All values were significantly greater than uninfected. Aliquots from the infected hDC samples were prepared by Diff-Quick staining and visualized by light microscopy. (**B**) The parasite index (#parasites/100 cells) and (**C**) the percentage of infected cells (%DC infected) is displayed. Mean values of individual donors ± SD are presented. *Statistical significance (p<0.05).

To explore the role of LPG on the IL12 response elicited from *L*. *major* infected hDCs, we quantified the relative amount of *IL12B* mRNA in hDCs after 8 hours of infection with FV1 WT, FV1 *lpg1* ^−^, and FV1 *lpg1* ^−^ */+LPG1* parasites. Compared to FV1 WT, FV1 *lpg1* ^−^ infected hDCs displayed a substantial decrease in IL12 expression (3.2 fold; Fig 2A) that was restored to levels approximately twice more than WT in the complemented FV1 *lpg1* ^−^ */+LPG1* line, perhaps consistent with a slight elevation of LPG in this line (S3C Fig, lanes 4 and 5). Our results indicate LPG plays a key role in IL12 induction in hDCs, consistent with the stimulatory effect seen with purified LPG (Fig 1B).

Conversely, FV1 *lpg2* ^−^ infected hDCs, relative to FV1 WT, displayed a significant increase in *IL12B* expression, that returned to comparable FV1 WT levels in the complemented FV1 *lpg2* ^−^ */+LPG2* line (Fig 2A). This observation was unexpected as FV1 *lpg2* ^−^ lacks LPG as well as other PGs, including PPGs (Fig 2C, lane 2). We considered the possibility that differences in infectivity between the WT and *lpg2* ^−^ could contribute to this result as *L*. *major* Lv39c5 *lpg1* ^−^ and *lpg2* ^−^ mutants exhibit reduced survival in peritoneal macrophages [41,59]. While parasite survival was slightly elevated in FV1 *lpg2* ^−^ infections, a comparable fraction of DCs were infected (Fig 2B and 2C), indicating, the differences observed in IL12 induction are likely not related to parasite survival in hDC under the conditions tested.

Thus, our studies showed that LPG is associated with increased IL12 production when tested biochemically (purified) or genetically (FV1 *lpg1* ^−^), while paradoxically *lpg2-* which also lacks LPG showed increased production. These data invoke the possibility *LPG2*-dependent molecules, such as phophoglycans including PPGs or other metabolites [60] may play a suppressive role on IL12 production. Alternatively, the loss of all *LPG2*-dependent structures may reveal another PAMP on the parasite surface that is able to induce IL12. Either scenario indicates a complex balance and interplay between parasite glycoconjugates and host cells.

**Fig 3:**
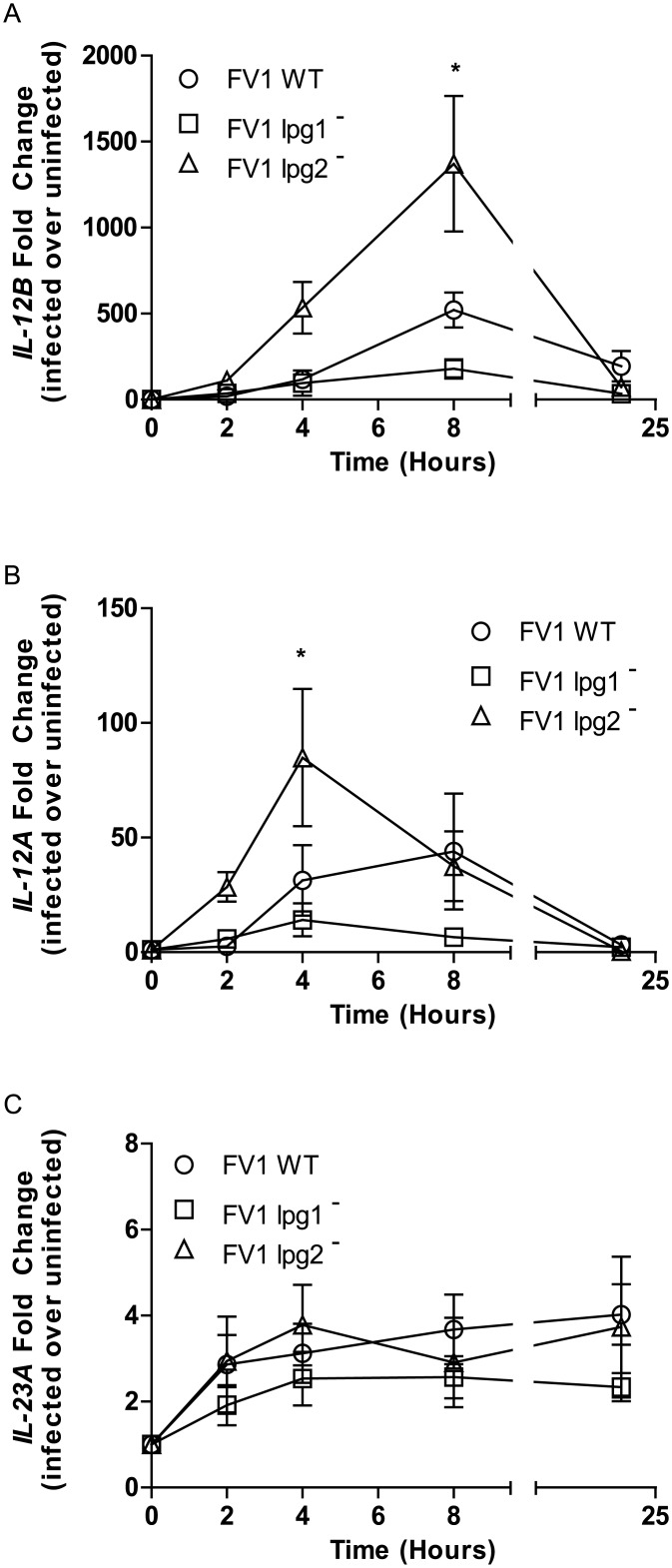
Kinetic analysis of *IL12* and *IL23* expression modulation by *L*. *major* LPG and PG null mutants. Human DCs (n = 3 donors) were infected with *L*. *major* FV1 parasites: *L*. *major* FV1 (WT), LPG null mutant (FV1 *lpg1* ^−^), LPG add back (FV1 *lpg1* ^−^/+*LPG1*), PG null mutant (FV1 *lpg2* ^−^), or PG add back (FV1 *lpg2* ^−^/+*LPG2*). At 2, 4, 8, and 24 hours post infection, *IL12B* (**A**), *IL12A* (B), and *IL23A* (C) expression was measured by qRT-PCR. Fold changes were calculated using the ΔΔC_T_ method and are represented as fold change over uninfected samples. Mean values of individual donors ± SD are presented. Statistical significance p<0.05, ANOVA with Bonferroni multiple comparisons test.

A kinetic analysis of these phenomena was conducted in DCs across four time points: 2, 4, 8, and 24 hours post-infection with FV1 WT and knockout mutants (Fig 3A). By 2 hours post-infection, FV1 *lpg2* ^−^ mutant infected hDCs induced slightly more *IL12B* compared with FV1 WT infected DCs. Albeit at higher expression levels than FV1 WT, FV1 *lpg2* ^−^ induced a similar kinetic *IL12B* mRNA response that declined by 24 hrs post infection. FV1 *lpg1* ^−^, on the other hand, induced little to no *IL12B* mRNA (Fig 3A). Similarly, FV1 *lpg2* ^−^ induced a quicker and more robust *IL12A* response compared to FV1 *lpg1* ^−^ and FV1 WT infections (Fig 3B). There were no differences between the WT and mutant strains for expression of the IL12 homolog *IL23A* (Fig 3C), suggesting that LPG and PGs regulate IL-12 production rather than IL-23.

### Human DC *TNF* expression is reduced during *L*. *major* FV1 *lpg1* ^−^ infections

**Fig 4:**
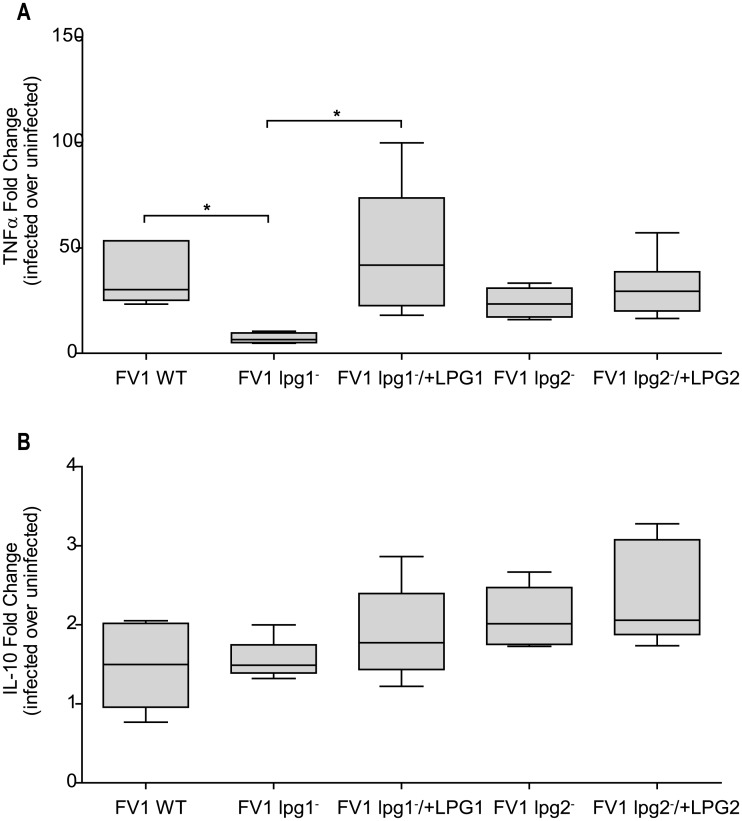
Relative *TNF* and *IL10* levels in *L*. *major* Friedlin V1 infected DCs. Human DCs were infected with *L*. *major* FV1 parasites: *L*. *major* FV1 *(WT)*, LPG null mutant (FV1 *lpg1* ^−^), LPG add back (FV1 *lpg1* ^−^/+*LPG1*), PG null mutant (FV1 *lpg2* ^−^), or PG add back (FV1 *lpg2* ^−^/+*LPG2*). At 8 hrs post infection, (**A**) *TNF* (n = 5 donors) and (**B**) *IL10* (n = 5 donors) expression was measured by qRT-PCR. Fold changes were calculated using the ΔΔC_T_ method and are represented as fold change over uninfected samples. Box plots display the median value (line), the interquartile range (box), and Tukey whiskers encompassing data within 1.5 fold of the interquartile range; data outside this range are presented as individual data points (open circles). *Statistical significance (p<0.05). All values were significantly greater than uninfected.

In addition to IL12, DCs are strong producers of other Th1 proinflammatory cytokines. TNF, for example, is significantly up-regulated in *L*. *major* infected hDCs [61]. We determined the relative fold induction of *TNF* in hDCs following infection with FV1 *lpg1* ^−^ and FV1 *lpg2* ^−^ mutants. We demonstrated that FV1 *lpg1* ^−^ induces significantly less *TNF* mRNA compared to WT or FV1 *lpg1* ^−^ */+LPG1* add back infections (Fig 4A), similar to the pattern of *IL12B* expression (Fig 2A). Infection with FV1 *lpg2* ^−^, however, was not statistically different compared to WT infection. The effect LPG has on both IL12 and TNF may contribute to the overall skewing of *L*. *major* towards a predominant Th1 response during cutaneous leishmaniasis.

### Down-regulation of *IL12B* in *L*. *major* FV1 *lpg1* ^−^ infection is not dependent upon IL10 induction

IL10 is generally implicated as a powerful inhibitor of IL12 production [62], and neutralizing IL10 promotes the ability of *L*. *major* parasites to establish IL12 production [63]. Here we quantified the *IL10* mRNA levels in hDCs infected with our mutant parasites to determine whether the failure of FV1 *lpg1* ^−^ to elicit sustained host IL12 induction relative to FV1 WT is due to the over-expression of IL10. The *IL10* expression elicited from hDCs infected with FV1 *lpg1* ^−^ or FV1 *lpg2* ^−^ did not differ from WT induced expression levels (Fig 4B), suggesting the mechanism by which these mutant parasites modulate *IL12B* expression is not dependent upon *IL10*.

### Human microarray analysis reveals broader gene expression effects of LPG and PPGs

**Fig 5:**
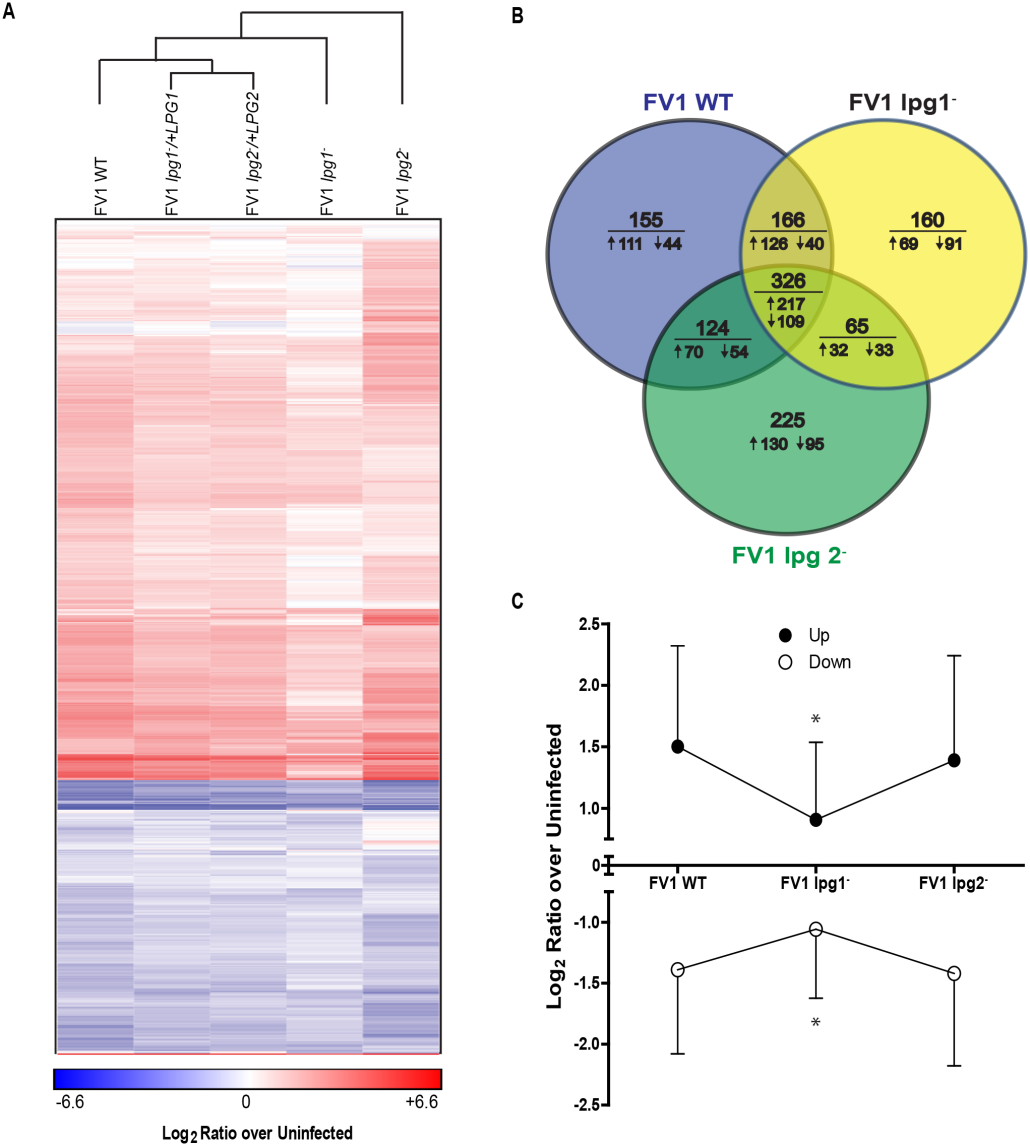
*Leishmania major* human host dendritic cells gene expression profiles. (A) Gene transcript expression heat map of *in vitro* infected monocyte-derived hDCs. The color scale is based on average log_2_ ratios of RMA-normalized microarray gene probe set values for variably infected host cells over uninfected cells. Only the genes that displayed significant differential expression by z-ratios, from both uninfected samples and between infections with FV1 WT and FV1 *lpg1* ^−^ or FV1 *lpg2* ^−^ mutants or their respective add back strains, were included in the map. Genes and sample types were clustered by city block distance metric using average linkage in GENE-E. (B) Venn diagrams with the number of host DC genes significantly differentially expressed from uninfected samples in FV1 WT, FV1 *lpg1* ^−^, and FV1 *lpg2* ^−^ mutants as quantified by microarrays. Values below the horizontal line indicate the number of genes from the above total that were up- (↑) or down-regulated (↓) compared to uninfected samples. (C) Total average log_2_ ratios of up- and down-regulated genes significantly differentially expressed from uninfected samples in FV1 WT, FV1 *lpg1* ^−^, and FV1 *lpg2* ^−^ mutants as quantified by microarrays, plus or minus standard deviation. *Significant difference of log_2_ ratio values (p<0.05) between FV1 *lpg1* ^−^ infected DCs compared to FV1 WT and FV1 *lpg2* ^−^ infected DCs by ANOVA with Bonferroni multiple comparisons test.

To further assess the influence of LPG and PPGs on host immunological responses, we infected additional DCs with *L*. *major* FV1 WT, mutants, and complemented strains, collecting mRNA at 8 hours post-infection. cDNA generated from these samples was hybridized to NimbleGen *Homo sapiens* Expression Microarrays. Expression of ten genes (*IL12B*, *IL1B*, *IL8*, *TLR4*, *TLR2*, *FKBP4*, *SOCS3*, *SMOX*, *FCGR1A*, and *TNFAIP3*) correlated significantly using qRT-PCR (p<0.000001, Spearman correlation coefficient = 0.784), validating the array values (S4 Fig). Gene transcript expression values were transformed to Z-scores and those genes that were significantly differentially expressed compared to uninfected cells (Z-score ≥ 1.96) were retained for downstream analysis. Hierarchical clustering of 730 genes that were expressed differently than FV1 WT infections in at least one mutant infection revealed that the complemented strains clustered more closely to the WT strains than their respective mutant strains (Fig 5A). Compared to uninfected cells, similar numbers of genes were regulated by infection with FV1 WT (771), FV1 *lpg1* ^−^(717) and FV1 *lpg2* ^−^ (740) (Fig 5B). Infection with FV1 WT resulted in more genes being up-regulated than either mutant strain (FV1 WT—524; FV1 *lpg1* ^-^—444; and FV1 *lpg2* ^−^—449). Notably, the magnitude of regulation (either up or down) was less during infection with FV1 *lpg1* ^−^ compared to either FV1 WT or *lpg2* ^−^ (Fig 5A and 5C), suggesting that this strain enters hDC in a silent fashion.

### LPG regulates immune response and infectious disease pathways

**Fig 6:**
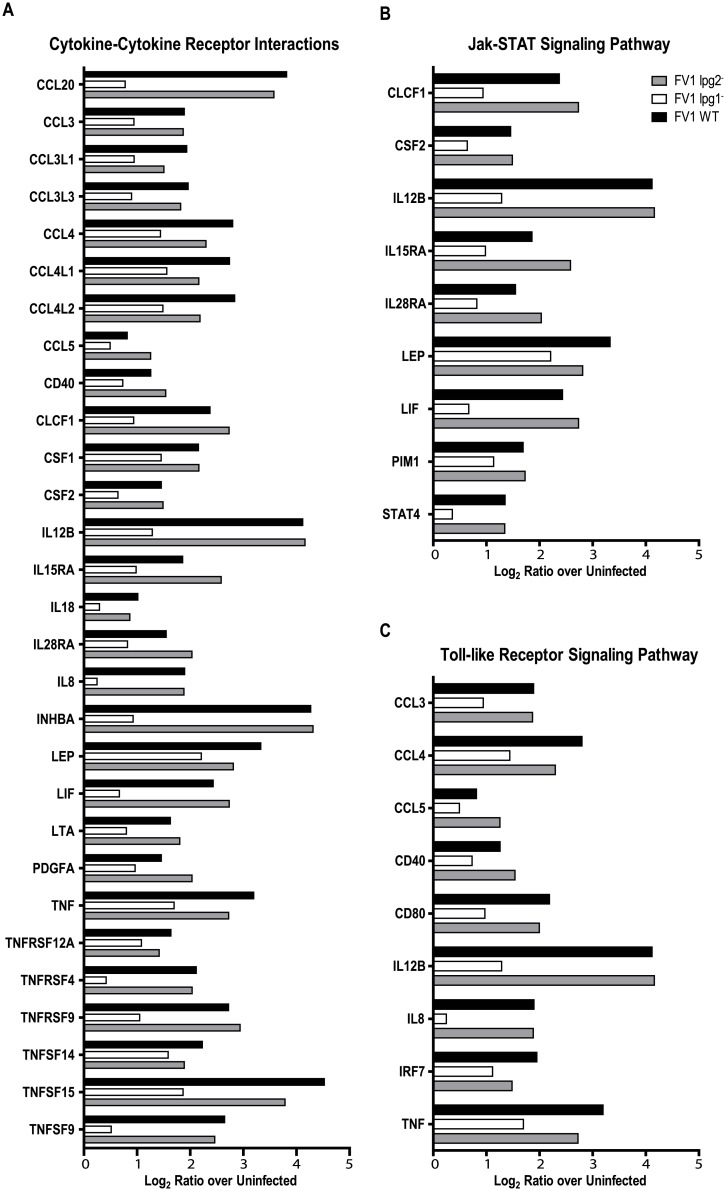
Enriched immunologically relevant pathways for genes expressed in *IL12B*-like patterns. Gene transcripts lists from *L*. *major* FV1 WT, FV1 *lpg1* ^−^, and FV1 *lpg2* ^−^ mutant *in vitro* infected monocyte-derived hDCs microarray analysis, with log_2_ ratio over uninfected value patterns between samples that clustered with the *IL12B*, were analyzed to identify significantly enriched (Benjamini and Hochberg adjusted p<0.01) KEGG pathways. Three immunologically relevant pathways, which also contained *IL12B*, were enriched among that gene list: cytokine-cytokine receptor interactions (A), Jak-STAT signaling pathway (B), and toll-like receptor signaling pathway (C). All graphs display average log_2_ ratio over uninfected values for genes present in the expression datasets of FV1 WT, FV1 *lpg1* ^−^, and FV1 *lpg2* ^−^ mutant infected samples which were members of the corresponding enriched pathways.

To assess the pathways involved in the regulation of IL12 by LPG, we utilized STEM and identified 233 genes that exhibited expression patterns similar to *IL12B* in response to infection with FV1 WT, FV1 *lpg1* ^−^, and FV1 *lpg2* ^−^. Overall *lpg2* ^−^ resembled WT while *lpg1* ^−^differed (Fig 6). Pathway enrichment revealed 22 significantly enriched pathways, mostly belonging to the immune response or infectious disease categories (Table 2). The most striking observation was the enrichment of three pathways: Cytokine-Cytokine Receptor Interactions, JAK-STAT Signaling and Toll-like Signaling, in which all the genes were down-regulated by infection with FV1 *lpg1* ^−^ compared to FV1 WT and FV1 *lpg2* ^−^ (Fig 6). Although the *lpg2-* pathway genes did not reflect any significance in this initial analysis compared to WT, future analysis of enriched pathways by criteria other than IL12 expression could reveal significant pathways enriched by *lpg2*- infection.

**Table 2:** Significantly enriched pathways for genes regulated similarly to *IL12B* by LPG.

Pathway Name	# Genes	p value
**Cytokine-cytokine receptor interaction**	29	2.31E-21
Rheumatoid arthritis	11	1.52E-08
**Toll-like receptor signaling pathway**	9	5.96E-06
Chagas disease (American trypanosomiasis)	8	5.22E-05
Amoebiasis	8	5.22E-05
**Jak-STAT signaling pathway**	9	8.16E-05
Cytosolic DNA-sensing pathway	6	8.16E-05
NOD-like receptor signaling pathway	6	8.16E-05
Chemokine signaling pathway	9	0.0003
Focal adhesion	9	0.0004
ECM-receptor interaction	6	0.0004
Allograft rejection	4	0.0011
Type I diabetes mellitus	4	0.0021
Intestinal immune network for IgA production	4	0.0032
Toxoplasmosis	6	0.0033
Hematopoietic cell lineage	5	0.0033
Malaria	4	0.0033
Small cell lung cancer	5	0.0033
Pathogenic Escherichia coli infection	4	0.0038
Phagosome	6	0.0061
African trypanosomiasis	3	0.0082
RIG-I-like receptor signaling pathway	4	0.0082

The most common transcription factor binding sites present in the promoters of genes regulated similarly to *IL12B* were identified using the DAVID functional annotation tool [56]. Not surprisingly, binding sites for transcription factor families known to regulate *IL12B* were identified, including, Octomer-binding transcription factor (OCT), Nuclear Factor Kappa B (NFκB), Interferon Regulatory Factor (IRF), cAMP Response Element Binding protein (CREB), and CCAAT/Enhancer Binding Protein families [64–68] (Table 3).

**Table 3:** Enriched Transcription Factor Binding sites in *IL12B*-like gene promoters.

Transcription Factor	# *IL12B*-like Genes	% *IL12B*-like Genes
AREB6	130	75.14
OCT1	128	73.99
AML1	123	71.10
CEBP	122	70.52
MEF2	113	65.32
NKX25	95	54.91
CDPCR3	80	46.24
GR	79	45.66
NFκB	78	45.09
RSRFC4	75	43.35
FOXO4	73	42.20
HMX1	72	41.62
ARNT	71	41.04
SOX9	71	41.04
COMP1	70	40.46
IRF7	70	40.46
MEIS1	70	40.46
CART1	68	39.31
NRSF	68	39.31
ELK1	67	38.73
RORA2	65	37.57
ARP1	64	36.99
CREBP1	63	36.42
NFKAPPAB	63	36.42
MEIS1BHOXA9	62	35.84
OCT	62	35.84
GFI1	61	35.26
IRF2	61	35.26
RORA1	61	35.26
HNF3B	60	34.68
NKX22	60	34.68
FOXO1	59	34.10
HAND1E47	59	34.10
HLF	59	34.10
P300	57	32.95
POU6F1	47	27.17
STAT5B	44	25.43

### Human DC *IRF8* expression is reduced during *L*. *major* FV1 *lpg1* ^−^ infection

**Fig 7:**
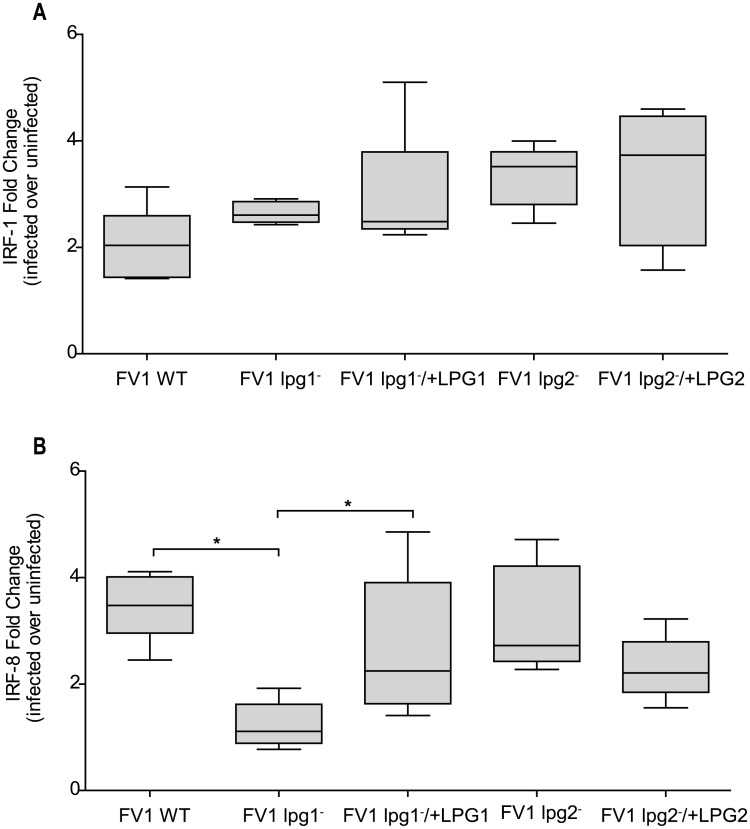
The *lpg1* ^−^ mutant affects IL12 associated gene regulator *IRF8*, and not *IRF1*. Human DCs were infected with *L*. *major* FV1 parasites: *L*. *major* FV1 *(WT)*, LPG null mutant (FV1 *lpg1* ^−^), LPG add back (FV1 *lpg1* ^−^/+*LPG1*), PG null mutant (FV1 *lpg2* ^−^), or PG add back (FV1 *lpg2* ^−^/+*LPG2*). At 8 hrs post infection, (**A**) *IRF8* (n = 3 donors) and (**B**) *IRF1* (n = 5 donors) expression was measured by qRT-PCR. Fold changes were calculated using the ΔΔC_T_ method and are represented as fold change over uninfected samples. Box plots display the median value (line), the interquartile range (box), and Tukey whiskers encompassing data within 1.5 fold of the interquartile range. *Statistical significance (p<0.05). All values were significantly greater than uninfected.

Production of IL12 relies on the nuclear translocation and cooperative binding of IRF-1 and IRF8 to IFNG-activated sequences (GAS) found within the *IL12B* promoter [18]. We previously demonstrated that *L*. *major* infection of hDC results in the early activation of NFκB transcription factors resulting in the transcriptional induction and nuclear translocation of IRF-1 and IRF-8 and, ultimately, IL12 production [42]. To delineate the effect of FV1 *lpg1* ^−^ and/or FV1 *lpg2* ^−^ on the upstream transcriptional features that regulate *IL12B* expression, we assessed *IRF1* expression in hDCs and observed that infection with FV1 mutants up-regulated *IRF1*, but not significantly more compared to WT induced levels (Fig 7A). This result suggests that the different *IL12B* responses displayed during FV1 *lpg1* ^−^ and FV1 *lpg2* ^−^ DC infections are not influenced by IRF1 expression. *IRF8* mRNA levels, however, were regulated by LPG. Infection with FV1 *lpg1* ^−^ resulted in a reduction of *IRF8* that is restored following infection with the FV1 *lpg1* ^−^ add back strain (Fig 7B). Infection with FV1 *lpg2* ^−^ did not significantly affect *IRF8* expression.

## Discussion

The major focus of this study was to investigate whether the enhanced IL12 immune response observed in *L*. *major* FV1 WT infected hDCs is dependent upon parasite LPG; as previous studies have implicated LPG plays a major role in modulating immune function in murine cells [31,69,70], as well as in human mononuclear cells [71–73]. First, we showed that, for this strain, infection with metacyclic promastigotes induces a high *IL12B* response (Fig 1B), compared to procyclic promastigotes and amastigotes, consistent with prior studies [57,58]. Additionally, we demonstrated that purified LPG stimulates an *IL12B* response in hDCs (Fig 1C). Similar studies utilizing purified *L*. *major* LPG from another strain have also highlighted the stimulatory effect LPG has on IL12 in human PBMCs [72].

To assess the role of surface molecules *in situ*, we employed genetic strategies to generate parasite mutants devoid of LPG (FV1 *lpg1* ^−^) or PG molecules and other LPG2-dependent metabolites (FV1 *lpg2* ^−^) in the *L*. *major* strain FV1 background (S3A Fig). Previous studies on the ‘low hDC IL12, *L*. *major* strain LV39c5 mutant parasites established several roles for LPG and PGs in regulating immune function [31–33,41,60]. For example, LV39c5 *lpg2* ^−^ induces IL12 in mouse BMDCs co-stimulated with anti-CD40 or IFNG [32,33]. In the absence of co-stimulation, however, there was no significant difference between IL12 elicited from LV39c5 WT or LV39c5 *lpg2* ^−^ parasites. We observed a similar result in our hDC assay where there was little difference in IL-12 induction between LV39c5 WT, LV39c5 *lpg2* ^−^, and LV39c5 *lpg2* ^−^ */+LPG2* infections (S6 Fig). Compared to FV1 WT, LV39c5 WT does not induce the same robust levels of *IL12B* (Fig 1A, S6 Fig).

Here, we generated *LPG1* and *LPG2* knockout mutants in the ‘high hDC IL12’ *L*. *major* FV1 background strain, in order to directly assess the parasite-derived molecular factors that contribute to the robust hDC IL12 response elicited by this strain of *L*. *major*. Our data demonstrated that the FV1 *lpg1* ^−^ mutant does not induce a high amount of *IL12B* transcript in hDCs as compared to FV1 WT (Figs 2A and 3A). Consistent with this observation, we showed that application of purified LPG was able to induce significant IL12 expression (S5 Fig), with both metacyclic *L*. *major* LPG which bears abundant PG side chain modifications, and *L*. *donovani* LPG, which is unmodified.

In contrast, and somewhat surprisingly given its similar lack of LPG, FV1 *lpg2* ^−^ up-regulates the *IL12B* response (Figs 2A and 3A) relative to FV1 WT. While in macrophage and animal infections the *lpg1-* and *lpg2*- mutants are typically attenuated [41,59], in our studies the survival of the WT and two mutant parasites did not differ significantly in DC survival over the course of these studies (Fig 2B and 2C). One explanation for this finding is that in *L*.*major* strain FV1, LPG and other *LPG2*-dependent glycoconjugates play inverse roles in stimulating the IL12 response in human DCs. One candidate for such an inhibitory LPG2-dependent molecule are the proteophosphoglycans (PPGs), which remain intact in the *lpg1* ^−^ mutant. Compared to LPG, little is known about the function of PPGs on host cell immune response, with evidence supporting roles as both an inhibitor or enhancer depending on the species and study [74–77]. PPGs vary structurally across species both in their PG and protein composition, and their large size and tendency to form polymeric aggregates renders their study more challenging [78]. Clearly, the development of mutants lacking only PPGs would be beneficial for future studies to directly assess the role these molecules have on the host cell response. Interestingly, amastigotes do not express significant amounts of the ‘pro-IL12’ LPG but do express high levels of PPG, which may further contribute to their inability to stimulate IL12 expression in hDCs. Importantly, the LPG2-dependent effect was also observed in the ‘low hDC IL12’ LV39 line, where ablation of LPG2 similarly resulted in increased IL12 production (S6 Fig)

Thus our data cause us to infer the presence of other LPG2-dependent PAMPs beyond LPG, with PPG as a possible candidate, and acting in an inhibitory fashion. The potential dominance of these inhibitory LPG2-dependent PAMPs provides an explanation for the conundrum that while all *Leishmania* species express LPG, despite that many do not induce IL12 [79]. Potentially, the strength of these suppressive LPG2-dependent PAMPs/processes may vary in different species and/or strains.

As it has been established that IRF1 and IRF8 are up-regulated in *L*. *major* infected hDCs and positively regulate *IL12B* gene expression [42], we assessed whether FV1 *lpg1* ^−^ or FV1 *lpg2* ^−^ affected the expression of *IRF1* and *IRF8*. Interestingly, FV1 *lpg1* ^−^ parasites caused a significant decrease in *IRF8* expression compared to WT (Fig 5A), indicating that LPG may influence the induction of *IL12B* by targeting upstream *IL12B* associated transcription factors that mediate its expression. Although IRF1 and IRF8 are known to cooperatively regulate *IL12B* gene transcription [42,80], we report that the FV1 *lpg1* ^−^ mutant does not affect *IRF1* expression compared to WT at 8 hours post-infection (Fig 4B). The distinct expression phenotypes exhibited by *IRF1* and *IRF8* following infection with FV1 *lpg1* ^−^ may be due to the difference in regulation of these two transcription factors. IRF1 is ubiquitously expressed, whereas IRF8 is preferentially expressed in immune cells and in response to activating signals. Furthermore, IRF1 and IRF8 can be differentially expressed in hDCs [81]. To bind target DNA sequences, IRF8 must bind to another transcription factor, compared to other IRF family members that can bind DNA sequences alone [82]. It is possible that infection with FV1 *lpg1* ^−^ reduces the amount of IRF8, which in turn inhibits the capacity of other transcription factors, such as IRF1, to form heterodimeric complexes that bind the *IL12B* promoter. These data suggest that LPG and not other PGs, enhance the *IL12B* response by a common mechanism involving *IRF8*.

Like IL12, *L*. *major* induces *TNF* in both human macrophages and DCs [61]. We therefore evaluated the relationship between parasite derived PG-bearing molecules on *TNF* using our LPG and PG null mutants. Our results demonstrate that the *lpg1* ^−^ mutant exhibits a significant decrease of *TNF* expression, similar to the reduction observed for *IL12B* (Fig 5A). Interestingly, the promoter regions for *IL12B* and *TNF* have similar transcription factor binding sequences, namely NFκB and ETS sites; the latter containing ISRE sequences that promote gene transcription upon IRF8 complex binding [83]. Therefore, it is possible that the reduction in *TNF* expression observed during FV1 *lpg1* ^−^ infection (Fig 5A) may also be IRF8-specific. A murine study demonstrated that cholera toxin (CT) inhibits plasmacytoid dendritic cellular IL12 by blocking the ability of IRF8 to bind to the ISRE sequence within the *IL12B* promoter, while IRF1 phosphorylation and subsequent binding to its DNA target sequence remained unaffected [84]. It is feasible that a similar mechanism exists in *L*. *major* infected cells, whereby IRF8 is specifically targeted for induction downstream of parasite LPG binding, subsequently leading to the induction of *IL12B* and *TNF*. Altogether, our data indicates that *L*. *major* FV1 skews the hDC response in an LPG-dependent manner towards a Th1-like polarization characterized by an increase in IL12 and TNF production which may be regulated by a common mechanism involving IRF8. A recent study demonstrated that macrophage induction of *IL12B* is controlled at the level of IRF8, which is specifically targeted for activation downstream of TLR4 in concert with Notch signaling pathways [85]. Interestingly, TLR4 [86] and other TLRs [87–91] have been implicated in recognition of parasite LPG.

An alternative explanation for the lack of an IL12 signal observed in the FV1 *lpg1* ^−^ infections may be a consequence of other functionally active PG-containing molecules, such as the PPGs which remain intact in the *lpg1* ^−^ mutant. These PPGs could provide an inhibitory IL12 signal. This theory is supported by our results demonstrating that FV1 *lpg2* ^−^, which lacks both LPG and PPGs, induces higher levels of IL12 compared to WT (Fig 2A), suggesting that some PG-containing molecules actually inhibit IL12 responses. In addition, amastigotes, on which LPG expression is drastically down-regulated and high levels of other PG containing glycoconjugates are highly expressed [15], do not induce IL12 (Fig 1B). Compared to LPG, little is known about the function of PPGs on host cell immune response. Previous work illustrating the ability of PPGs to induce complement activation by triggering the mannose binding protein pathway [76] and their inability to elicit CD4^+^ T-cell response in murine bone marrow derived macrophages [74], concludes that PPGs may contribute to the chronic infections observed during *L*. *mexicana* infections. However, it has been demonstrated that *L*. *major* PPGs require IFNG priming to induce TNF and NO production in murine macrophages [77]. In human PBMCs, PPGs cause an induction of IL10 and to a lesser extent NO and IL12 [75]. Although these studies provide conflicting implications for PPGs role as either inhibitor or enhancer of immune response, it is difficult to compare studies because the repertoire of PPGs structure varies across species [78]. Additionally, the use of purified PPGs can be problematic because the amount of purified PPGs added is often higher than what is biologically present during an actual infection, therefore the development of mutants lacking only PPGs would be beneficial for future studies to directly assess the role these molecules have on the host cell response. We measured *IL-10* mRNA levels in our mutant-infected DCs, because of the generally inhibitory effects of IL-10 on IL12 [62]. However, IL10 expression exhibited between FV1 *lpg1* ^−^, FV1 *lpg2* ^−^, and WT infected hDCs did not differ (Fig 5B), ruling out one theory that the decrease in *IL12B* expression observed during FV1 *lpg1* ^−^ could be consequence IL10 overproduction. Another explanation for the induction of IL12 by FV1 *lpg2* ^−^, is the possibility that the absence of all surface and secreted PGs reveals a molecular pattern or some other molecule that induces IL12.

Our microarray analyses of FV1WT, FV1 *lpg1* ^−^, and FV1 *lpg2* ^−^ infected hDCs revealed that FV1 *lpg1* ^−^ enter hDC in a relatively silent fashion as indicated by the overall down-regulation of significantly expressed transcripts, (Fig 6), and the overall reduction in genes belonging to cytokine and TLR related gene pathways, (Fig 7). Altogether these data suggest that a lack of LPG molecules results in silent entry and that LPG is a major pattern recognized by pattern recognition receptors on DCs. As with the IL12 response, the absence of all PGs appears to either release some sort of repression or reveals a molecular pattern that compensates for the lack of LPG, highlighting the complexity of DC pattern recognition receptor interactions in controlling host responses to *Leishmania* infection. Future analyses focusing on FV1 *lpg2-* mutant infections may reveal pathways uniquely regulated by PGs.

This work adds to the growing set of genetically modified parasites (*lpg1* ^−^, *lpg2* ^−^ in the *L*. *major* FV1 background) providing biologically relevant tools for assessing the role of parasite surface glycoconjugates on cellular function in human and mouse model systems, as well as, provides insight into the complex interplay of LPG and other PG molecules on the cellular immune response elicited following *L*. *major* infections by global gene expression analyses.

## Supporting Information

S1 Fig Confirmation of *L*. *major* FV1 *lpg1* ^−^ mutant. (**A**) Schematic representation of WT (top) and *lpg1* ^−^ alleles (bottom). Numbers represent primers used for PCR amplification. PCR analysis for one representative FV1 WT, FV1 *lpg1* ^−^ (cl 2.10) and FV1 *lpg1* ^−^ */+LPG1* (cl 2.10 AB3) is depicted. (**B**) Primers 1/2 (SMB1023/SMB1626) confirmed *LPG1* disruption: WT (420bp), FV1 *lpg1* ^−^ (3200bp) and FV1 *lpg1* ^−^ */+LPG1* (420bp & 3200bp). (**C**) Primers 3/11 (SMB4183/SMB2566), and 12/6 (SMB4185/SMB4184) established the integration of the 5’ flanking (3300bp) and 3’ flanking (2600bp) sequences of the *LPG1*::*HYG* disruption cassette, respectively. Primers 3/7 (SMB4183/SMB2889), and 8/6 (SMB2888/SMB4184) established the integration of the 5’ flanking (3400bp) and 3’ flanking (2800bp) sequences, of the *LPG1*::*PAC* disruption cassette, respectively. Primers 9/10 (SMB2891/SMB2892) and 4/5 (SMB1568/SMB1569) confirmed the presence of the *HYG* (1080bp) and *PAC* (600bp) ORFs, respectively.
(EPS)

S2 Fig Confirmation of *L*. *major* FV1 *lpg2* ^−^ mutant. (**A**) Schematic representation of WT (top) and *lpg2* ^−^ alleles (bottom). Numbers represent primers used for PCR amplification. PCR analysis for one representative FV1 WT, FV1 *lpg2* ^−^ (cl 6.1A) and FV1 *lpg2* ^−^ */+LPG2* (cl 6.1A AB15) is depicted. (**B**) Primers 13/14 (SMB1023/SMB1626) confirmed replacement of *LPG2*: WT (1000bp), FV1 *lpg2* ^−^ (absent) and FV1 *lpg2* ^−^ */+LPG2* (1000bp). (**C**) Primers 15/11 (SMB4124/SMB2566), and 19/20 (SMB2565/SMB4125) established the integration of the 5’ flanking (1300bp) and 3’ flanking (2200bp) sequences of the *LPG2*::*HYG* replacement cassette, respectively. Primers 15/17 (SMB4124/SMB3507), and 16/18 (SMB3506/SMB4417) established the integration of the 5’ flanking (1700bp) and 3’ flanking (2400bp) sequences, of the *LPG2*::*SAT* replacement cassette, respectively. Primers 9/10 (SMB2891/SMB2892) and 16/17 (SMB3506/SMB3507) confirmed the presence of the *HYG* (1080bp) and *SAT* (600bp) ORFs, respectively.
(EPS)

S3 Fig Generation of *L*. *major* FV1 LPG null mutant (FV1 *lpg1* ^−^) and PG null mutant (FV1 *lpg2* ^−^) mutant. WT parasites underwent two rounds of electroporation as described in the methods to generate the FV null mutants. (**A**) For FV1 *lpg1* ^−^, WT parasites were transfected with *^LPG1*::*HYG* and screened heterozygotes underwent a 2^nd^ round of transfection with *^LPG1*::*PAC* to yield FV1 *lpg1* ^−^ null mutant. A third round of transfection with add back vector pXG-*LPG1*::*NEO* restored *LPG1*. (**B**) The FV1 *lpg2* ^−^ was created utilizing a similar transfection strategy with targeting constructs, Δ*LPG2*::*HYG*, and Δ*LPG2*::*SAT*. The *LPG2* was restored by electroporation with add back vector pSNBR-*LPG2*::*NEO*. (**C**) Western blot analysis with anti sera WIC79.3 indicated a loss of LPG in the FV1 *lpg1* ^−^ mutant (lane 6) and loss of both PPGs and LPG in the FV1 *lpg2* ^−^ mutant (lane 2). The FV1 *lpg1* ^−^/+*LPG1* add backs (two clones are shown, lane 4,5) and the FV1 *lpg2* ^−^/+*LPG2* add back (lane 3) exhibit restored levels of LPG and PPGs, comparable to WT parasites (lane 1).
(EPS)

S4 Fig Validation of microarray expression by qRT-PCR. Ten genes were selected for validation by qRT-PCR analysis (A)*IL12B*, (B) *SOCS3*, (C) *TNFAIP3*, (D) *IL1B*, (E) *IL8*, (F) *TLR4*, (G) *TLR2*, (H) *FKBP4*, (I) *SMOX*, and (J) *FCGR1A*. For each gene, fold change was calculated using fold changes were calculated using the ΔΔC_T_. Log_2_ ratios of RMA-normalized microarray gene probe set values for infected host cells over uninfected cells from the microarray (black bars) and fold changes from the qRT-PCR analysis (gray bars) are plotted and subjected to Pearson Correlation test (R value). Mean ± SEM is presented.
(EPS)

S5 Fig Purified LPG stimulates *IL12B* expression in hDCs. Human DCs were exposed to 1 μg LPG derived from *L*. *major* or *L*. *donovani* promastigotes (n = 3 donors). After 8 hours, RNA was extracted from infected hDCs for cDNA generation and analyzed for *IL12B* expression by qRT-PCR. Fold change was calculated utilizing the ΔΔC_T_ method and depicted as fold change over uninfected samples. Mean ± SEM is presented.
(EPS)

S6 Fig *L*. *major* LV39c5 induced hDC IL12 responses do not differ between LV39c5 mutants. Human DCs (n = 4 donors) were infected with *L*. *major* parasites: *L*. *major* FV1 WT (FV1), *L*. *major* LV39c5 WT (LV39c5), LV39c5 PG null (LV39c5 *lpg2* ^−^), and LV39c5 PG add back (LV39c5 *lpg2* ^−^ */+LPG2*). At 8 hrs post infection, *IL12B* expression was measured by qRT-PCR. Fold change was calculated utilizing the ΔΔC_T_ method and depicted as fold change over uninfected samples. Box plots display the median value (line), the interquartile range (box), and Tukey whiskers encompassing data within 1.5 fold of the interquartile range. *Statistical significance as compared to uninfected control, (p<0.05). All values were significantly greater than uninfected.
(EPS)

S1 Table Primers used for molecular generation of *L*. *major* FV1 mutants. (DOCX)

S2 Table Human Primer Sequences for qRT-PCR analysis. (DOCX)
