# Supplementary material for: Differential Impact of LPG-and PG-Deficient Leishmania major Mutants on the Immune Response of Human Dendritic Cells
Source: PLoS Negl Trop Dis. 2015 Dec 2;9(12):e0004238. doi: 10.1371/journal.pntd.0004238 (PMC4667916; doi:10.1371/journal.pntd.0004238)
Supplement: S1 Table — (DOCX) [file pntd.0004238.s007.docx]

| **Table S1. Primers used for molecular generation of *L. major* FV1 mutants** | |
| --- | --- |
| primers to confirm *L. major* FV1 *lpg1^-^* & FV1 *lpg1^-^/+LPG1* | |
| SMB 1023 | AGATCTCGGGTCTGGCACGTTTTTCAGCG |
| SMB 1626 | TAATACCGGTGACGATGGATTA |
| SMB 4183 | GCCTTTCTTCTCCGACCCTTCTGA |
| SMB 1568 | TTAGATGGATCCTCAGGCACCGGGCTTGCG |
| SMB 1569 | TATGATACTAGTATGACCGAGTACAAGCCCAC |
| SMB 4184 | CCGCTGCACGTGAGGGTTCTGT |
| SMB 2889 | ACCGTGGGCTTGTACTCGG |
| SMB 2888 | ACGTCGAGTGCCCGAAGGAC |
| SMB 2891 | GGAGGACCCGGGCCACCATGAAAAAGCCTGAACTCACCG |
| SMB 2892 | GGAGGATCTAGACTATTCCTTTGCCCTCGGACGA |
| SMB 2566 | GAAAGCACGAGATTCTTCGC |
| SMB4185 | GCGACATCGAACAGGACGGAATAG |
| SMB 2567 | CGTTGGCTACCCGTGATATTGC |
| SMB 2728 | TCAGAAGAACTCGTCAAGAAGG |
| primers used to construct plasmid B6598 | |
| SMB 4120 | AAGCTTCGTCGACGTGTGCACTCCTCTC |
| SMB 4121 | CGTCAGCCCGCACCGTTACCGACAGTTGCTGATGCAATTCTTTCGG |
| SMB 4122 | GCACCTTACGTGGGATCTCGCACCGTTGTTAGCCGCAGTCGTAG |
| SMB 4123 | CTCGAGGTTACAGCAATGCGCCCACTCA |
| SMB 4077 | GGTAACGGTGCGGGCTGACGCCACCATGAAGATTTCGGTGATCCCTG |
| SMB 4078 | CGAGATCCCACGTAAGGTGCTTAGGCGTCATCCTGTGCTCCC |
| primers to confirm *L. major* FV1 *lpg2^-^* and FV1 *lpg2^-^/+LPG2* | |
| SMB 2527 | GCGCGGATCC ATGAACCACACTCGCGCTGTCATGGAGG |
| SMB 2528 | GCCCAAGCTTCTACTCAGATTTGAAGTTGTCACTGCTG |
| SMB 4124 | GGCGACGTTCTCTTCTCTTGTATC |
| SMB 3506 | CATCCCCGGGATGAAGATTTCGGTGATCCCTG |
| SMB 3507 | CATCCCCGGGTTAGGCGTCATCCTGTGCTCCC |
| SMB 4417 | TCACAGAACCAAACCGCC |
| SMB 2891 | GGAGGACCCGGGCCACCATGAAAAAGCCTGAACTCACCG |
| SMB 2892 | GGAGGATCTAGACTATTCCTTTGCCCTCGGACGA |
| SMB 2566 | GAAAGCACGAGATTCTTCGC |
| SMB 2565 | AATACGAGGTCGCCAACATC |
| SMB 4125 | GCACGACGCCCACATCC |
| SMB 2559 | GGTAACGGTGCGGGCTGACGCCACCATGGGATCGGCCATTGAACAAG |
| SMB 2560 | CGAGATCCCACGTAAGGTGCTCAGAAGAACTCGTCAAGAAGG |
