# Supplementary material for: Differential Impact of LPG-and PG-Deficient Leishmania major Mutants on the Immune Response of Human Dendritic Cells
Source: PLoS Negl Trop Dis. 2015 Dec 2;9(12):e0004238. doi: 10.1371/journal.pntd.0004238 (PMC4667916; doi:10.1371/journal.pntd.0004238)
Supplement: S2 Table — (DOCX) [file pntd.0004238.s008.docx]

| **Table S2.** Human Primer Sequences for qRT-PCR analysis | | |
| --- | --- | --- |
| **Gene** | **Forward** | **Reverse** |
| *FCGR1A* | CCTTTGGGTTCCAGTTGAT | CCTCACAGTGCAAGGTTAC |
| *FKBP4* | AGGCAGTCCTCCAAAGAT | TCACCGCGAGTCTGTATT |
| *HPRT* | ACACTGGGAAAACAATGGAGA | AAGCTTGCGACCTTGACCAT |
| *IL1B* | TTCTTCGACACATGGGATAACG | TGGAGAACACCACTTGTTGCT |
| *IL8* | TGCGCCAACACAGAAAT | TTATGAATTCTCAGCCCTCTTC |
| *IL10* | TCCTTGCTGGAGGACTTTAAGGGT | TGTCTGGGTCTTGGTTCTCAGCTT |
| *IL12A* | ATGCTCCAGAAGGCCAGACAAACT | TCCAATGGTAAACAGGCCTCCACT |
| *IL12B* | TGGATGCCGTTCAGAAGCTCA | TGGACCTGAACGCAGAATGTCA |
| *IRF1* | AGAGCAAGGCCAAGAGGAAGTCAT | ACTGTGTAGCTGCTGTGGTCATCA |
| *IRF8* | AGAAGAGCATGTTCCGGATCCCTT | ACAGCGTAACCTCGTCTTCCAAGT |
| *SMOX* | ACTTCTTGAGCAGGGTTTC | TGGCGTGTCCAAGTTTC |
| *SOCS3* | AGAGCCTATTACATCTACTCCGGG | GGGTGACTTTCTCATAGGAGTCCA |
| *TLR2* | TGCAAGCAGGATCCAAAG | TGCTTCAACCCACAACTAC |
| *TLR4* | AAGCCGAAAGGTGATTGTTG | CTGAGCAGGGTCTTCTCCAC |
| *TNF* | TGGGCAGGTCTACTTTGGGATCAT | TTTGAGCCAGAAGAGGTTGAGGGT |
| *TNFAIP3* | AGTGTTCCCAGGTGGCCTTAGAAA | TCTCAGCCAAGACGATGAAGCAGT |
